# Tender X-ray diffraction anomalous fine structure spectroscopy applied to the study of PbSc_0.5_Nb_0.5_O_3_ relaxor ferroelectric oxide

**DOI:** 10.1107/S1600577525007428

**Published:** 2025-09-17

**Authors:** G. Ciatto, Y. Bing, Z.-G. Ye, P.-E. Janolin

**Affiliations:** a Synchrotron SOLEIL, L’Orme des Merisiers, Saint-Aubin, BP 48, F-91192Gif sur Yvette Cedex, France; b Department of Chemistry and 4D LABS, Simon Fraser University, 8888 University Drive, Burnaby, BC, V5A 1S6, Canada; c Laboratoire SPMS, UMR CNRS-CentraleSupélec, Bâtiment Gustave Eiffel – MB.105, 8–10 rue Joliot-Curie, 91190Gif sur Yvette Cedex, France; University of Malaga, Spain

**Keywords:** diffraction anomalous fine structure spectroscopy, tender X-ray, relaxor ferroelectric oxides, superstructure reflections, chemical order

## Abstract

Diffraction anomalous fine structure spectroscopy (DAFS) is a well established technique for characterizing the local structure of elements embedded in complex interfaces and templates when crystallographic and/or site selectivity is needed. DAFS has been effectively applied in the hard X-ray range, where reciprocal space is extended and the absorption edges of the chemical elements are usually well spaced. In this work, we extend the use of DAFS to the tender X-ray range. This energy range is important since it includes the *L*-edges of second row transition metal elements, which are constituents of functional oxide materials; and some important edges for semiconductors. We present a study of the Nb *L*-edges in PbSc_0.5_Nb_0.5_O_3_ relaxor ferroelectric oxide, where the use of superstructure reflections provides access to the ordered part of the sample.

## Abstract 2

At the SIRIUS beamline of Synchrotron SOLEIL, the use of the DAFS technique has been extended to the tender X-ray range. We present a study of the Nb *L*_3_-edge in PbSc_0.5_Nb_0.5_O_3_ relaxor ferroelectric oxide, where the use of superstructure reflections provides access to the ordered part of the sample.

## 1. Introduction

Diffraction anomalous fine structure spectroscopy (DAFS) (Sorensen *et al.*, 1994 ▸; Renevier *et al.*, 2003 ▸) is a synchrotron based structural characterization technique belonging to the resonant elastic X-ray scattering (REXS)/resonant X-ray diffraction (RXRD) family (Vettier, 2012 ▸; Hodeau *et al.*, 2001 ▸). During a DAFS experiment the maximum intensity of an X-ray diffraction (XRD) Bragg peak is followed using an X-ray diffractometer, while scanning the energy of the incident photons across an absorption edge, similar to what is done in X-ray absorption spectroscopy (XAS) experiments (Lee *et al.*, 1981 ▸). DAFS spectra exhibit a fine structure after the absorption edge, related to the complex nature of the atomic scattering factor, which encodes information on the neighborhood of the absorber, similarly to those accessible via XAS. In contrast to REXS/RXRD, which focus on the study of the resonance and where short spectra are acquired across the absorption edge, DAFS employs longer spectra (from several tenths of electronvolts up to several hundred electronvolts after the edge when possible) to investigate the fine structure. In contrast to XAS, in DAFS the local structural information is related to the subset of atoms that make up the long-range order that produces the selected XRD peak. DAFS combines the chemical selectivity of XAS and the crystallographic sensitivity of XRD, thereby establishing itself as a unique analytical tool for the study of complex interfaces, epitaxial templates, mixtures or crystals in a matrix. DAFS has been used, for example, to determine the short-range order about a single atomic type in a sample of mixed amorphous and nanocrystalline phases of germanium (Frenkel *et al.*, 2002 ▸) to address the composition and strain in III–V semiconductor nanostructures (Favre-Nicolin *et al.*, 2012 ▸; Proietti *et al.*, 1999 ▸; Ciatto *et al.*, 2005 ▸), and the structure of magnetic nanocrystals in a semiconductor matrix (Navarro-Quezada *et al.*, 2021 ▸). The variation in crystallographic weights of atoms across different sites, which change when measuring different Bragg reflections, can also yield site selectivity. This approach has been successfully employed, for instance, in the determination of the local structure surrounding octahedrally and tetrahedrally coordinated Fe atoms in magnetite (Frenkel *et al.*, 1999 ▸). More recently, DAFS has been applied to investigate the interchanging of transition metals in battery electrodes (Kawaguchi *et al.*, 2015 ▸; Kawaguchi *et al.*, 2014 ▸) and to study magnetoelectricity and multiferroics, coupled with anisotropy in anomalous scattering (Zschornak *et al.*, 2014 ▸).

DAFS began to be recognized as a structural characterization tool in materials science in the 1980s and 1990s following the development of synchrotron radiation sources. It was demonstrated to be successful primarily in the hard X-ray range (Stragier *et al.*, 1992 ▸; Pickering *et al.*, 1993 ▸), where the reciprocal space is extended, the absorption edges of the chemical elements are well spaced and the experimental setup often does not require a vacuum chamber. The vast majority DAFS experiments described in the referenced articles and reviews above (and references therein) were conducted in the hard X-ray range. Concurrently, REXS was established as a key technique in the soft X-ray range to investigate magnetic, orbital and charge ordering phenomena related to electronic degrees of freedom (Fink *et al.*, 2013 ▸). Moreover, it was recently identified as a promising tool in polymer science (Collins & Gann, 2022 ▸). In this context, the tender X-ray range (1–5 keV range) has been largely neglected by DAFS experiments, despite the fact that the first historical observation of the diffracted intensity energy dependence was made on a peak of mica, around the Al *K*-edge (1.56 keV) (Cauchois, 1956 ▸). To the best of our knowledge, some DAFS experiments were carried out at the *M*_3_-, *M*_4_- and *M*_5_-edges of U (3.4 to 5.0 keV) in uranium arsenide on charge and magnetic reflections to probe the strong orbital contribution to the magnetic moment (McWhan *et al.*, 1990 ▸). Additionally, DAFS has been utilized in soft matter, around the S *K*-edge (2.47 eV), in the study of liquid crystals (Mach *et al.*, 1998 ▸; Mach *et al.*, 1999 ▸) and conjugated polymers (Freychet *et al.*, 2021 ▸). However, the analysis was limited to only 20 eV after the absorption edge.

We strongly believe that the use of DAFS in the tender X-ray range should be much more widely adopted in materials science research, despite the more challenging technical requirements. The *L*-edges of the second row of transition elements (Sr, Y, Zr, Nb, Ru, Mo *etc*.), the building blocks for many functional oxide materials, all belong to the tender X-ray range, along with several other edges of semiconductor constituents (Al, Si, P, In, Sb *etc*.). Furthermore, the *K*-edge of S and the *L*-edges of Mo are particularly relevant for the study of transition metal dichalcogenide 2D materials such as MoS_2_ and TiS_2_ (Venkata Subbaiah *et al.*, 2016 ▸). The interest in employing *L*_3_- and *L*_2_-edges for transition metal atoms arises from the fact that they probe transitions from *p* to *d* orbitals, hence the *d*-partial local density of non-occupied states. *L*_3_- and *L*_2_-edge XAS and DAFS are expected to be more sensitive to variations in the local symmetry and bond distortions since they probe valence orbitals. The high sensitivity of Nb *L*_3_- and *L*_2_-edge spectra to the local site symmetry and the electronic configuration around Nb atoms, along with the usefulness of Nb *L*_2_–*L*_3_-edge XAS spectroscopy for Nb speciation in minerals, have been already demonstrated by other groups (Bollaert *et al.*, 2023 ▸). The choice of the Nb *L*-edges for an exemplar tender X-ray DAFS experiment is pertinent since they are located close to the middle of the tender X-ray range.

In this study, leveraging our high-vacuum diffractometer endstation (Ciatto *et al.*, 2019 ▸), we utilized tender X-ray DAFS to study PbSc_0.5_Nb_0.5_O_3_ functional oxide. This material, which is characterized by a complex perovskite structure, may exhibit either ferroelectric or relaxor behavior depending on the ordered or disordered distribution of Nb and Sc cations on the crystal lattice sites, in bulk (Chu *et al.*, 1995 ▸; Isupov, 2003 ▸) as well as in thin film form (Janolin *et al.*, 2008 ▸; Tyunina *et al.*, 2013 ▸). By choosing a superstructure reflection generated by the chemical order of the cations to record the Nb *L*_3_-edge DAFS spectra, we are able to select the subset of resonant scatterers arranged in the corresponding ordered structure. DAFS taken on a superstructure reflection is compared with DAFS on a structure reflection and with XAS recorded on the same sample. The experimental DAFS and XAS data are then compared with *ab initio* simulations, which are based on the insights of neutron diffraction (ND) experiments and calculations.

## 2. Experimental and theoretical methods

### 2.1. Sample growth

The PbSc_0.5_Nb_0.5_O_3_ single crystals were grown by a high-temperature solution method (Guo *et al.*, 2012 ▸; Bing & Ye, 2003 ▸; Bing & Ye, 2006 ▸). Because of the high melting point of the material, the use of a flux was necessary in order to lower the growth temperature. High-purity (>99.99%) powders of PbO, Sc_2_O_3_ and Nb_2_O_5_ were used as starting materials. The stoichiometric starting powders were directly mixed with the complex flux of PbO/B_2_O_3_ (an excellent solvent for this application) before starting the pre-melting and heating steps. We refer to the references given above for details on the sample growth. The domain structure of the samples was investigated by TEM (Nakajima *et al.*, 2025 ▸).

### 2.2. Tender X-ray DAFS/XAS setup

DAFS experiments were performed at the Nb *L*_3_-edge (approximately 2371 eV) at the SIRIUS beamline of Synchrotron SOLEIL (Ciatto *et al.*, 2016 ▸), using the multilayer grating monochromator (MGM) and the tender X-ray high-vacuum diffractometer (Ciatto *et al.*, 2019 ▸). The beamline, which is mounted on an HU36 undulator source, provides a high flux of photons on the sample in the tender X-ray range. With this configuration, the flux is of the order of 10^13^ photons s^−1^ with open experimental slits. The energy resolution (Δ*E*/*E*) of the MGM, used with an energy selection slit closed at 5 µm, is between 1.5 × 10^−4^ and 2 × 10^−4^ in the tender X-ray range. Even if slightly worse than that of our double crystal monochromator (DCM), this energy resolution allows us to set an energy step of 0.3–0.5 eV at the Nb *L*-edges, which is more than sufficient for XAS and DAFS spectroscopies. In general, we prefer to use the MGM in the 2–2.5 keV energy range because its mechanics are more stable during the energy scans, since they work with smaller optical angles (maximum 3.5° for the scans shown in this work). With the Si(111) DCM we would need to select large Bragg angles for energies close to 2 keV. The beam was focused only in the vertical plane using a C-coated mirror with mechanical benders. XAS was also performed in conjunction with DAFS at the same beamline. XAS was recorded in fluorescence mode using an in-vacuum four-element silicon drift detector (Bruker XFlash QUAD 5040), while DAFS was measured using an in-vacuum PILATUS3 100 K-M 2D detector.

To address both the crystal structure and chemical order on the crystal lattice sites (see below), the DAFS measurements were carried out around the (002) and (111) crystal plane reflections. In the experiments shown here, we set a slit of 0.5 mm × 0.2 mm (V × H) before the sample, resulting in a flux at the sample of about 3.5 × 10^12^ photons s^−1^ when no attenuator was used. We note that the measurements around the (111) reflection were conducted without the use of attenuators, while we had to use a 20 µm-thick Al attenuator to protect the 2D detector when measuring the (002) structural reflection; in this case the flux on the sample was reduced to (1–5.5) × 10^10^ photons s^−1^ along the (002) DAFS scan. The collection time for a single XAS and DAFS spectrum was 3 s per point. The estimated number of photons at the 2D detector at the maximum of the XRD spot was 2.7 × 10^4^ photons s^−1^ pixel^−1^ for the (002) reflection and 1.1 × 10^4^ photons s^−1^ pixel^−1^ for the (111) reflection. The total time for a full XAS and DAFS spectrum like those presented in Section 3 was about 18 min including the acquisition deadtime in step-by-step mode. However, for the (111) reflection, we summed ten scans to compensate for the lower integrated XRD intensity with respect to the (002) one and to maintain roughly the same signal-to-noise level for all the spectra presented.

Note that accessing the (002) and (111) reflections of the crystals under study in the tender X-ray is not trivial. The former requires access to rather large detector angles (2θ > 82° in the present case), while the latter (non-specular) necessitates an in-vacuum four-circle diffractometer geometry, as a refractometry setup is insufficient. Synchronization of the beamline undulator energy, monochromator energy and diffractometer angles was performed using a software script implementing Bragg’s law while scanning in step mode. Normalization of the XAS and DAFS spectra was carried out using a prototype ultra-thin optical-grade diamond membrane specifically designed for monitoring the beam intensity and position and for normalizing spectra in the tender X-ray range (Desjardins *et al.*, 2014 ▸).

### 2.3. Tender X-ray DAFS/XAS data reduction and *ab initio* simulation

**Figure 1:**
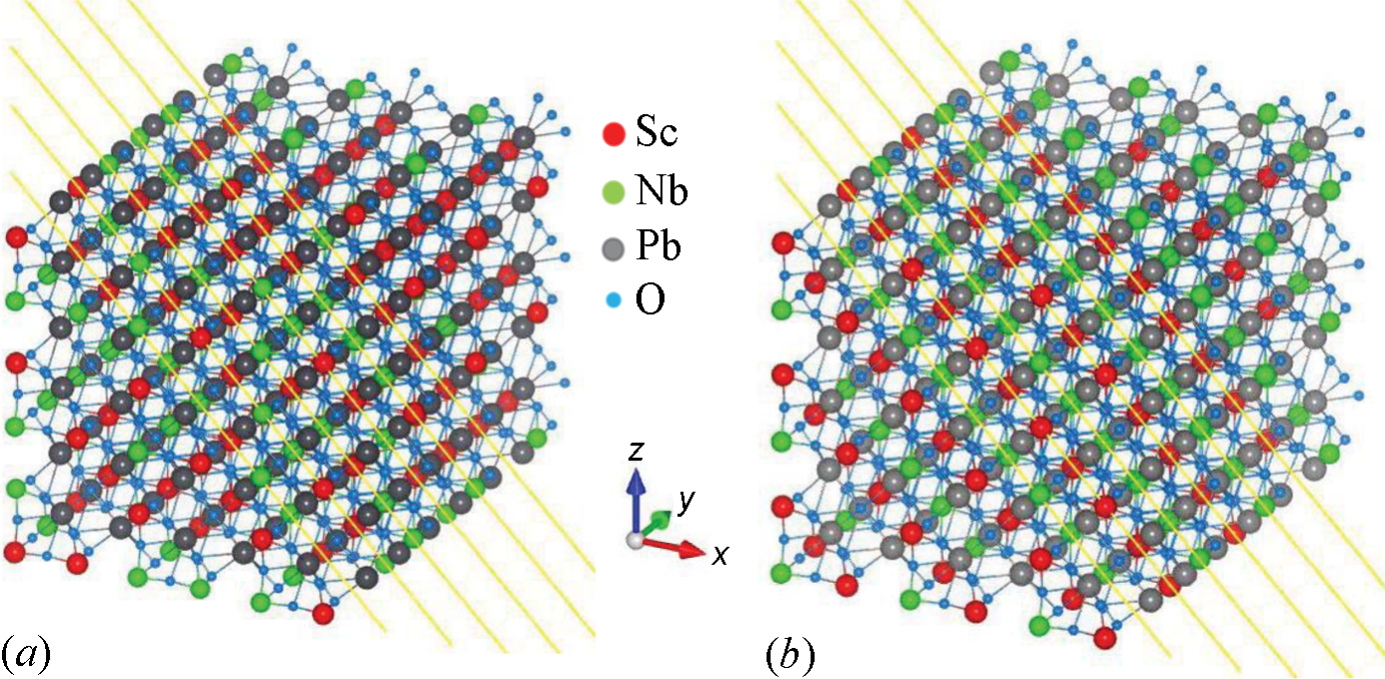
Representation of the two model supercells used in the simulations, simplified by showing 1080 atoms only. (*a*) Disordered supercell. (*b*) Ordered supercell. A [121] projection vector (⊥ to [111]) was used in the view to highlight the stacking of the (111) planes, the upwards vector was [001]. Note that in the disordered structure all crystal planes ⊥ to [111] can contain both Nb and Sc atoms (that are randomly distributed), whereas in the ordered one these planes contain either Nb or Sc atoms, in strict alternation moving along [111].

The XAS data were corrected by self-absorption (Pfalzer *et al.*, 1999 ▸) and non-linearity (Ciatto *et al.*, 2004 ▸) while all necessary corrections (absorption, Lorentz, polarization, geometrical, filters) were made on the DAFS data (Renevier *et al.*, 2003 ▸). The XAS and DAFS spectra were simulated using the *FDMNES* code (Bunău & Joly, 2009 ▸) used in the Green function full multiple scattering approach. Two configurations of 5000-atom clusters (corresponding to supercells composed of 800 perovskite *AB*O_3_ unit cells) were created using the same lattice parameters, differing in the arrangement of 400 Nb and 400 Sc atoms on the ‘*B*’ site of the perovskite structure. The lattice parameters used were determined from single-crystal ND. In the first configuration, the atom occupying each ‘*B*’ site was chosen randomly while maintaining an equal total number of Nb and Sc atoms. Such configuration corresponds to a disordered structure. In the second configuration, Nb and Sc atoms were placed on alternating (111) planes, resulting in a perfect chemical order. Note that Pb always occupies the *A* site of the cell. Fig. 1 ▸ shows the two atomic configurations used in the simulations. Spheres of 20 Å radius were cut from the original cluster around a central Nb atom, and XAS and DAFS were calculated around each Nb atom inside the sphere, using clusters of 9 Å radius around the absorber for each calculation and averaging the spectra from all absorber sites. Concerning the parameters used in the *FDMNES* calculation, the ‘Gamma hole’ parameter was fixed to 1.66 eV, corresponding to the natural width of the Nb *L*_3_ level given in the tables by Krause & Oliver (1979 ▸). The convolution parameters for the energy-dependent broadening were *Ecent* = 35, *Elarg* = 20 and *Gamma max* = 20. As for the experimental broadening, we used a Gaussian function with a convolution width of 1 eV, slightly higher than the theoretical energy resolution of the monochromator, compensating for the fact that the clusters used in the simulations do not consider possible small local distortions of the interatomic distances related to the mixed cation configuration. The same parameters were used for all simulations: XAS and DAFS, ordered and disordered models.

## 3. Results and discussion

**Figure 2:**
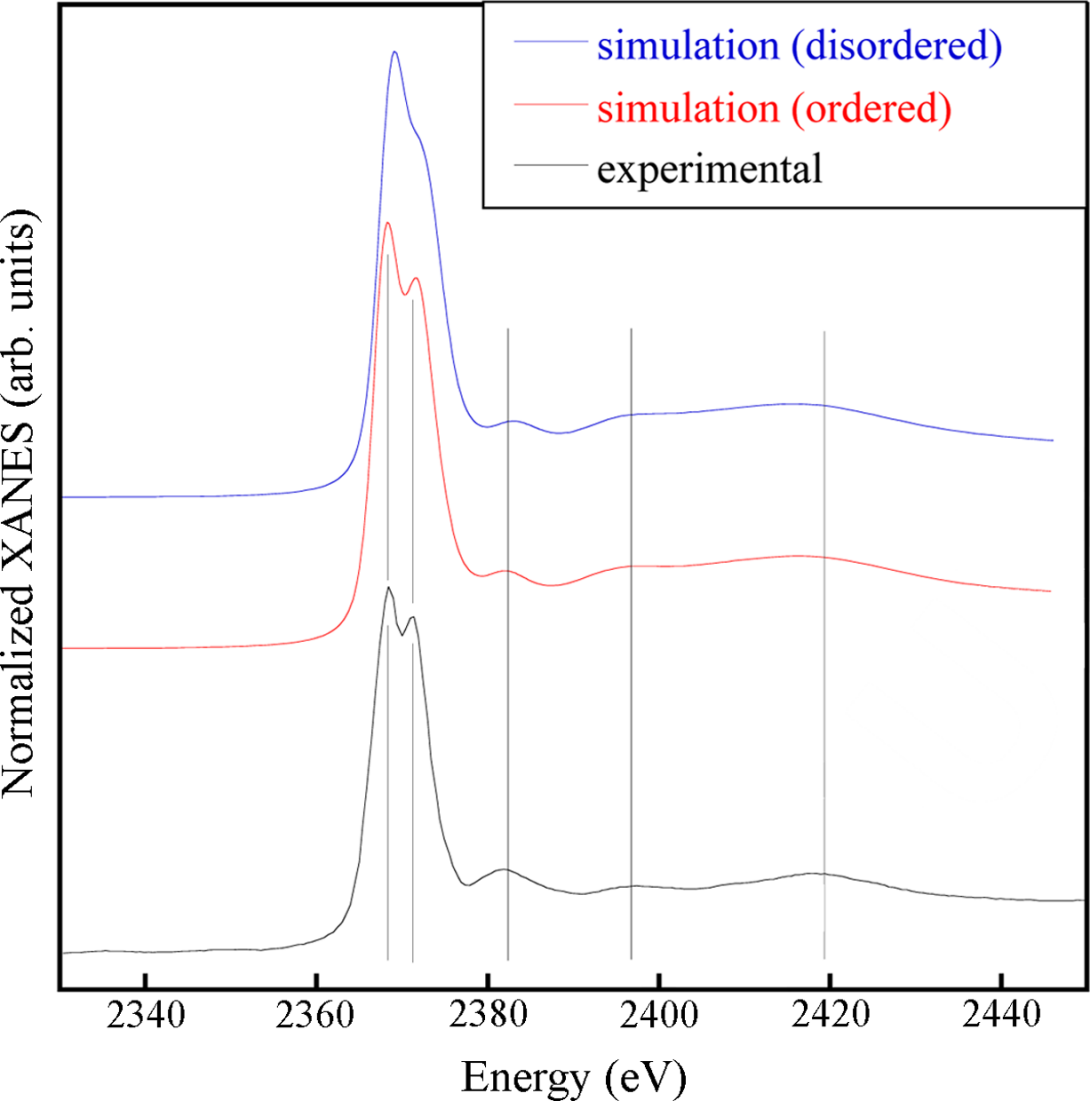
Nb *L*_3_-edge XAS spectrum (bottom line) compared with *ab initio* simulations based on model clusters mimicking perfect Nb/Sc order on the *B*-site (middle line) and random Nb/Sc distribution, *i.e.* disorder, on the *B*-site (top line). The vertical lines are guides to the eye, indicating the main feature positions.

Fig. 2 ▸ shows the Nb *L*_3_-edge XAS spectrum obtained for the PbSc_0.5_Nb_0.5_O_3_ sample (bottom line) and compares it with *ab initio* simulations, obtained as described above, corresponding to the ordered (middle line) and disordered (top line) cation distributions. The experimental spectrum shows two peaks just after the white line, at *E* = 2368 eV and 2371 eV. The simulation for the *B*-site ordered cation distribution reproduces this doublet and all other spectral feature positions. Conversely, the simulation for the disordered distribution falls short in reproducing the doublet, as it only captures the first peak, while the second peak manifests itself as a shoulder. In a molecular orbital picture, the two strong peaks forming the doublet can be attributed to the dipole-allowed transitions from the Nb 2*p*^3/2^ level to empty 2*t*_2*g*_(π*) and 3*e*_*g*_(σ*) molecular orbitals (Bera & Yusuf, 2021 ▸; Sugiura *et al.*, 1988 ▸). The two peaks are associated with the Nb 4*d* states and their separation (Δ) with the ligand-field splitting of the 4*d* states of octahedral Nb. Although Nb is always in octahedral coordination for both the disordered and ordered configurations, the separation and shape of the two peaks may be sensitive to the degree of distortion of the octahedron, charge transfer and other factors. The experimental Δ observed was found to be 2.9 eV, close to the values observed for NH_4_NbF_6_ and Nb_2_O_5_ (Sugiura *et al.*, 1988 ▸), and is consistent with the splitting of Nb^5+^ coordinated to oxygen ligands in weakly distorted octahedra (Galoisy *et al.*, 1999 ▸).

**Figure 3:**
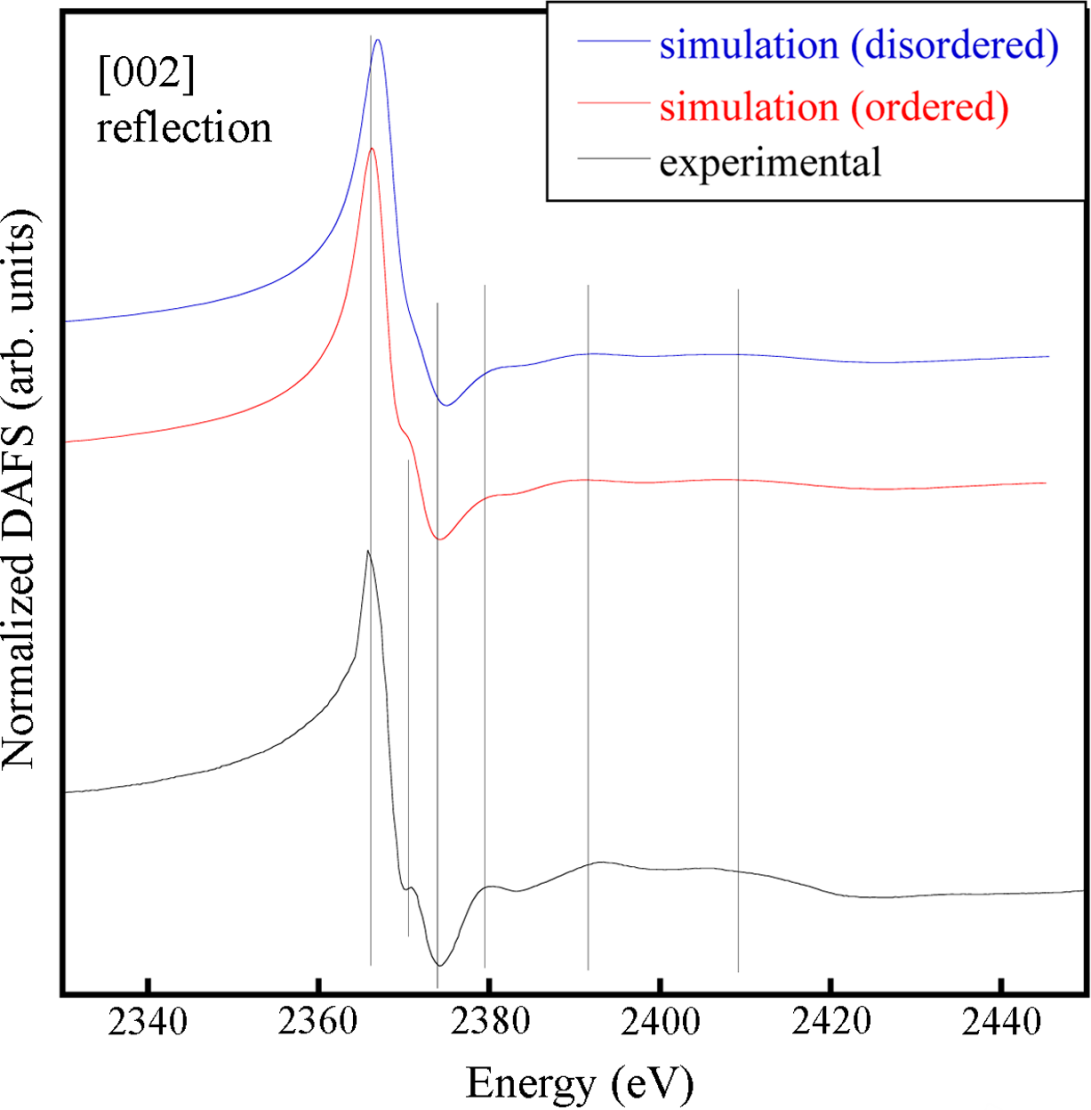
Experimental and simulated Nb *L*_3_-edge DAFS spectra for the same configurations at the (002) structural reflection. The vertical lines are guides to the eye, indicating the main feature positions.

A similar outcome is obtained when observing the Nb *L*_3_-edge DAFS experimental and simulated spectra around the (002) crystal plane reflection (Fig. 3 ▸). (002) is a structural reflection, meaning that all atoms comprising the crystal participate in its structure factor. As a consequence, it is supposed to provide information on the average local structure of the crystal around Nb atoms, similar in this case to the one available from XAS. The experimental DAFS spectrum shows a shoulder around 2370 eV, which is also present in the simulated spectrum for the ordered configuration, while it is absent in the simulation of the disordered configuration.

Note here that, as mentioned above, the two models used for simulating the XAS and DAFS spectra are created using the same lattice parameters and differ only in the distribution of the cations on the *B*-site. Despite the fact that no distortion of the octahedra is considered in the models, the simulated spectra show a very different shape in the near-edge region, with the ‘ordered’ configuration producing a much clearer doublet. This suggests that the observed doublet structure is more related to a configurational effect, related to the presence of cation ordering rather than to distortion of the interatomic distance distribution. In a (full) multiple scattering picture, which is the approach used in our simulations, the spectral region very close to the absorption edge is sensitive to configurational ordering of the neighbors even in the middle-range since the photoelectron free mean path can reach a few tens of ångstroms. Nb and Sc are very different atoms, with the former having more than double the atomic weight than the latter, and different occupancy of valence *d* states. The backscattering amplitude of the two atoms in the multiple scattering process are also sensibly different. Therefore, the atomic/interatomic potential felt by the photoelectron will be different in the case of a different distribution of the two cations even in the ‘theoretical’ case of no variation of the distance distribution and in the ‘physical’ case of small variation. We can guess that, in the case of a more ordered distribution of the cations, the different possible multiple scattering events can sum up more efficiently due to the increased symmetry, producing clearer spectral resonances. Conversely, in the case of random distribution of cations, the increased configurational disorder can induce some broadening of the multiple scattering features.

The conclusion of an ordered configuration of Nb/Sc cations on the *B*-sites based on the analysis of the XAS and (002) DAFS spectra is consistent with preliminary XRD and ND results on the same sample, which suggest a close to perfect order on the *B*-site of the perovskite (Janolin *et al.*, 2025 ▸). However, note that, from a quantitative perspective, the differences in the spectra are not substantial enough to preclude the presence of a small fraction of disordered PbSc_0.5_Nb_0.5_O_3_ based solely on the XAS results. It can be reasonably inferred that, for a specimen exhibiting a more pronounced mixture of the two configurations, the assessment of the order degree from the experimental spectrum would have been more intricate, and the deconvolution of the ordered portion of the specimen would have been unfeasible.

**Figure 4:**
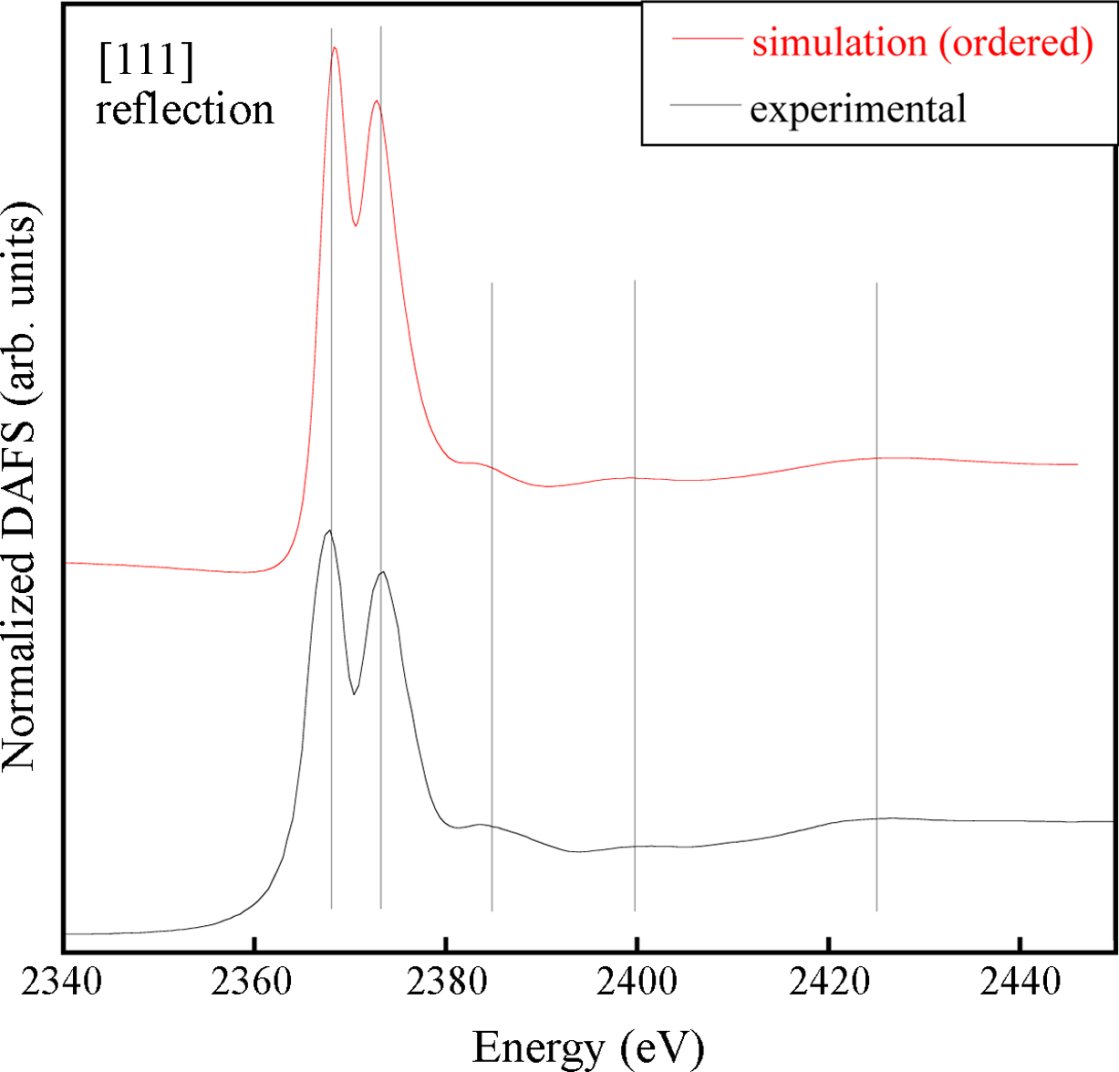
Nb *L*_3_-edge DAFS spectrum taken at the (111) chemical reflection (bottom line) compared with an *ab initio* simulation based on a perfect Nb/Sc order on the *B*-site. The vertical lines are guides to the eye, indicating the main feature positions.

**Figure 5:**
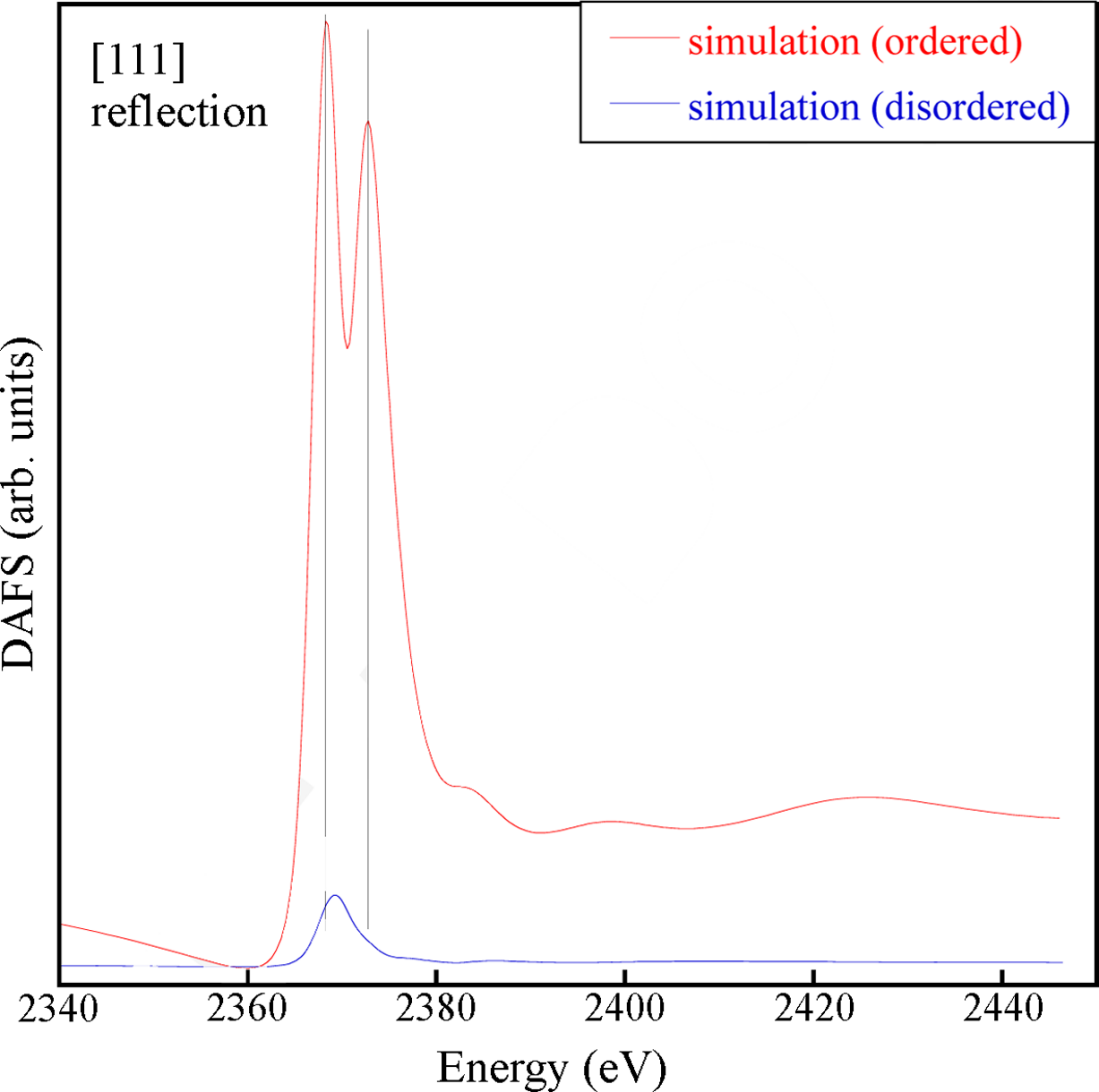
Nb *L*_3_-edge simulated (111) DAFS spectra, assuming a perfect Nb/Sc order and random occupation on the *B* site. The vertical lines are guides to the eye, indicating the main feature positions.

Fig. 4 ▸ shows the Nb *L*_3_-edge DAFS spectrum measured around the (111) reflection (bottom line). In contrast to the (002) one, the (111) reflection is referred to as a ‘chemical’ reflection, because it originates from the chemical order on the *B* site. This indicates that only the subset of resonant atoms generating this order in the crystal contributes to the production of the DAFS signal. The spectrum shows a much more distinct doublet, with peaks centered at *E* = 2368 eV and 2373 eV. This observation lends further credence to the hypothesis that the observed doublet structure is closely associated with the presence of cation ordering in the system. Notably, a simulation assuming an ordered configuration on the *B*-site can accurately reproduce the spectral feature positions and amplitudes. Furthermore, the enhanced definition of the doublet facilitates a more precise comparison between the experimental spectrum and simulations based on different models; in this instance, it provides an unequivocal validation of the cluster model used to emulate the cation ordering configuration (see Section 2.3). In the case of strong distortion of the local symmetry and important variations of the cation local environment from that of the model, we would not have found such good agreement with the experimental data. Fig. 5 ▸ presents non-normalized simulated (111) DAFS spectra derived from the ordered and disordered clusters. It is evident that in the simulation for the disordered cluster the doubled peak is completely absent. Moreover, the intensity of the simulated signal is significantly lower than the one simulated for the ordered cluster. This outcome is consistent with the notion that the (111) diffraction peak originates from the ordered arrangement of the cations in the crystal planes perpendicular to [111]. Therefore, the ‘disordered’ simulated DAFS signal in Fig. 5 ▸ is generated by statistical randomness only.

This observation demonstrates the potential of DAFS in discerning the environment of an absorber embedded within different structures and its crystallographic selectivity. In instances where the sample or material exhibits a lower degree of chemical order relative to the one under study, DAFS on the (111) reflection would have continued to provide insights into the local structure and the local density of unoccupied states within the ordered subset of absorbers. This approach would enable the discrimination of these ordered regions from the disordered part of the sample. With respect to other techniques that can be used for the determination of the degree of ordering at the *B* site, such as powder XRD and ND, the near-edge region of DAFS (as for XAS) provides spectral features which are sensitive to the local symmetry and related to the local density of non-occupied states of materials. Such information on the local environment and electronic structure is out of the reach of pure diffraction techniques. In the case of variations and distortions of the local symmetry and of the cation local environment, DAFS and XAS would be sensitive to such variations and an insight of the local structure could be retrieved via *ab initio* modeling of the modified structures and spectra simulations, similar to those presented in this paper. Unlike XAS, DAFS can provide information on the local and electronic structure of the ordered part of the sample, which can be separated from the rest.

The tender X-ray DAFS experimental station utilized in this study is fully accessible for users at the SIRIUS beamline of Synchrotron SOLEIL and it has been recently used in several other experiments for the study of more complex materials, such as solid solutions between antiferroelectric PbYb_0.5_Nb_0.5_O_3_ and ferroelectric PbTiO_3_ (Sanchez *et al.*, 2025 ▸), GeSbTe phase change materials (Daoudi *et al.*, 2025 ▸) and SrVO_3_ based quantum wells (Dumont *et al.*, 2025 ▸). The analysis of the high-quality DAFS data collected in this energy range is currently underway.

## 4. Conclusions

At the beamline SIRIUS of Synchrotron SOLEIL, the DAFS technique has been expanded to encompass the tender X-ray range, a feat made possible by the distinctive instrumentation available at this facility. This energy range has not been extensively explored by DAFS due to technical challenges; however, it encompasses several absorption edges of interest for modern materials science. In this study, we employed tender X-ray DAFS performed around the *L*_3_-edge of Nb (around 2371 eV) to investigate the chemical ordering of the cations in PbSc_0.5_Nb_0.5_O_3_ relaxor ferroelectric oxide. This ordering plays a major role in determining the dielectrical properties of the materials. By selecting a superstructure reflection (111) arising from the chemical order of the cations for the recording of the DAFS spectra, we were able to select the subset of Nb scatterers arranged in the corresponding ordered structure. A comparison of the experimental XAS and DAFS spectra with *ab initio* simulations suggests that the sample is close to full chemical ordering of Nb/Sc cations on the *B*-site of the perovskite structure, which is in agreement with complementary characterizations. Note that the results presented are merely the initial example of a series of successful tender X-ray DAFS experiments performed at the beamline, whose data are currently under analysis.
